# Nutrition Assistance Programs and Pediatric Weight Outcomes: A Systematic Review

**DOI:** 10.3390/nu18030394

**Published:** 2026-01-25

**Authors:** Dan Ferris, Genevieve Davison, Tyler Frank, Amanda Gilbert, Fanice Thomas, Sydney Rothman, Kim Lipsey, Sarah Moreland-Russell

**Affiliations:** 1 Brown School, Washington University in St. Louis, St. Louis, MO 63130, USA; dan.ferris@wustl.edu (D.F.);; 2 Department of Psychiatry, Center for Healthy Weight and Wellness, School of Medicine, Washington University in St. Louis, St. Louis, MO 63110, USA; genevieve.davison@wustl.edu; 3 School of Public Health, Washington University in St. Louis, St. Louis, MO 63130, USA; tylerfrank@wustl.edu (T.F.); a.s.gilbert@wustl.edu (A.G.); 4 Bernard Becker Medical Library, School of Medicine, Washington University in St. Louis, St. Louis, MO 63110, USA; lipseyk@wustl.edu

**Keywords:** food security, nutrition assistance programs, school meals, body mass index, pediatric obesity, policy interventions

## Abstract

Background/Objectives. Food insecurity and pediatric obesity have increased concurrently in the U.S., raising questions about the role of Federal Nutrition Assistance Programs (FNAPs) in shaping weight outcomes. This systematic review examined evidence on relationships between FNAP participation and pediatric weight outcomes. Methods. Six databases were searched for U.S.-based, peer-reviewed studies published through July 2024 that assessed FNAP participation and pediatric weight outcomes. Results. Seventy-five studies met the inclusion criteria, and no consistent pattern indicated that any single FNAP or program type (educational setting-based or direct financial support) reliably reduced or increased childhood overweight or obesity risk. Twenty studies found statistically significant beneficial relationships between FNAP participation and pediatric weight outcomes. Most studies reported mixed findings (*n* = 32), typically varying by subgroup (e.g., age, grade level, gender, race or ethnicity, or program characteristics). Sixteen studies found no relationship between participation and weight. Seven studies found an adverse relationship. Most studies relied on non-randomized quantitative designs and secondary data, and adverse findings were more common in lower quality studies. Among 18 studies that evaluated the effects of policy changes (e.g., the Healthy Hunger-Free Kids Act (2010), 2009 WIC package change), nearly all identified associations between the policy change and weight outcomes, with eight beneficial and nine reporting mixed results. Conclusions. The findings indicate a complex non-causal relationship between FNAP participation and weight that varies across populations, programs, and study designs. Overall, evidence does not support broad adverse weight effects of FNAPs, and policy changes that strengthen nutrition standards may contribute to healthier weight outcomes. These findings have implications for nutrition policy, program design, and future research.

## 1. Introduction

In 2022, 6.4 million (17.3%) U.S. households with children under age 18 experienced low or very low food security (i.e., food insecurity), defined as the lack of “access by all people at all times to enough food for an active, healthy life” [1]. In more than half of these households (3.3 million), both children and adults experienced food insecurity, resulting in 13.4 million children living in food insecure households [2]. In 2022, the prevalence of food insecurity in households with children was significantly higher than in both 2020 and 2021 [2]. Food insecurity incidence was disproportionately higher in households with annual incomes below 100% to 185% of the federal poverty level and among individuals identifying as Black, Hispanic, or Native American [2].

Research shows that child food insecurity results from systemic social, economic, and political factors that limit the resources needed to access and afford healthy food [3]. Families experiencing poverty, unemployment, and income shocks are more likely to experience food insecurity. The high cost of food, especially healthy and nutritious food, as well as spending on other necessities (e.g., utilities, healthcare, housing), compounds the strain on household resources needed to afford food [3]. Other factors such as distance from or availability of affordable healthy foods exacerbate increased food insecurity among children [3].

Along with rising food insecurity rates, overweight and obesity have increased among children between the ages of two and 19 years old. Childhood obesity prevalence (20%) has more than tripled since the late 1970s, with disproportionate impacts on young Black and Hispanic children and children with low socioeconomic status [4]. Around 25% of Black children and 26% of Hispanic children have obesity, compared to 17% of White children [5,6]. Twenty-six percent of children living in households with incomes below 130% of the federal poverty level have obesity [5,6]. Further, obesity increases with age [5,6]. In 2020, 13% of children aged two to five years had obesity, compared to 21% of school-aged six-to-11-year-old children, and 22% of 12–19-year-old children [5,6].

Left unaddressed, childhood obesity and overweight can persist into later ages [7], with some estimates indicating that up to 90% of children and adolescents retain or experience increased overweight and obesity in adulthood [8,9]. These rates are significant for health and healthcare as obesity is associated with serious health conditions and outcomes, including cardiovascular disease, metabolic syndrome, disability, and mortality. In addition to the health impact, overweight, obesity, and associated chronic disease cost an estimated $1.7 trillion annually in medical costs and lost productivity [10]. Given the risks and high costs of childhood obesity, understanding both potential contributors and protective mechanisms is of critical importance for policy, practice, and research [11,12].

Food insecurity and obesity prevalence have simultaneously increased, a trend that researchers have termed the “food insecurity-obesity paradox” [13,14]. Food insecurity has been identified as an obesity risk factor in children [4] and adults [15]. Of youth experiencing food insecurity, 42% are overweight or have obesity [16]. Various theories have sought to identify and explain links between food insecurity and obesity: the caloric density of lower-cost foods most likely to be available to individuals experiencing food insecurity [17], environmental and geographic considerations such as food deserts and food swamps [18], behavioral reactions to scarcity and uncertainty around a basic need such as food [19,20,21], and the targeting of advertisement and marketing of unhealthy products [22]. The cyclical relationship between food insecurity and chronic disease, including obesity, has also been explored in previous research [4,23,24].

A population-level strategy for addressing food insecurity among children is Federal Nutrition Assistance Programs (FNAPs). FNAPs include a variety of policies and programs that aim to improve food access through (1) the provision of food in institutional or educational settings (e.g., National School Lunch Program, School Breakfast Program) or (2) household financial supports or resources that families can use to purchase food. Although most predate the emergence of ‘food security’ as a measured construct, they have always sought to improve food access for targeted individuals and families experiencing short- or longer-term financial hardship [25]. Overall, research has found that these programs succeed at their primary stated purpose of reducing food insecurity and increasing food access [26,27,28]. However, ongoing debate surrounds the relationship between FNAPs and childhood overweight and obesity, with some suggesting FNAPs may even contribute to rising obesity rates among children.

Large-scale FNAPs that increase child food security include the Supplemental Nutrition Assistance Program (SNAP) and the Special Supplemental Nutrition Program for Women, Infants, and Children (WIC). These programs provide financial resources for individuals and families to purchase food items. SNAP reaches around 15 million children nationwide, 20% (3 million) of whom also receive WIC [29]. WIC increases access to healthy food for 6.4 million pregnant, postpartum, and breastfeeding individuals and their children up until age 5 [5]. SNAP does not have national nutritional requirements, whereas WIC provides food items that meet specific nutritional guidelines and household needs [30]. WIC also provides nutrition and breastfeeding education [30].

Beyond SNAP and WIC providing financial assistance directly to families, other FNAPs increase food access through institutional or educational settings, where children and adolescents spend significant time (e.g., private and public schools, daycare centers, after-school programs) and where many children receive a significant source of their nutrition [29]. Found to decrease child hunger and improve nutrition, these programs include the National School Lunch Program (NSLP), the School Breakfast Program (SBP), and the Child and Adult Care Food Program (CACFP). Food provided through FNAPs in educational settings must meet nutritional guidelines [31]. These programs have significant reach, with the NSLP providing meals for 29.4 million children and SBP feeding 15.4 million children in the 2023–2024 school year [5,32]. In the fiscal year 2024, the NSLP, SBP, CACFP, and Summer Food Service Program served 9.3 billion meals [33].

While FNAPs have been found to reduce food insecurity in children, there is ongoing debate around the role FNAPs play in childhood overweight and obesity. FNAPs have been cited as potential strategies for preventing obesity in children, especially among children in lower-income households or those living in poverty [5,34]. However, various interests have attempted to connect, dispute, or contextualize the rise in obesity with FNAP participation. Some studies connect increased obesity prevalence with participation in FNAPs [35,36,37,38], while others have found no significant relationship between obesity and nutrition assistance programs, or even beneficial outcomes such as reduced childhood obesity [39,40] or reduced low birth weight and rapid infant weight gain [41].

Researchers have conducted numerous literature reviews related to food insecurity as well as nutrition assistance programs and their effects [28,42,43,44]. Previous systematic reviews indicate significant research interests in food security and nutrition [45], program and policy interventions [46,47,48], and weight status [49]. None, however, has looked systematically at relationships between FNAPs with pediatric weight change indicators. One notable recent study of global food insecurity interventions focused on nutrition intervention evaluations rather than potential relationships with health or weight outcomes. Their study findings highlight mixed observations in relation to food security intervention participation and weight, and that research has examined process outcomes, employed non-randomized study designs, and relied heavily on parental and self-report metrics [50]. Other reviews have provided valuable insights on specific topics such as food pantry participation [51] or benefits of Meals on Wheels programs [52]. A PROSPERO registry search revealed two registered studies with similar aims but with different identified geographies and no indication of advancing past registration (CRD42016052850 and CRD42015025055).

Addressing this research gap can show: (1) which types of FNAPs (e.g., financial support through WIC and/or SNAP; food provision through educational settings) reduce or contribute to childhood obesity; (2) when during childhood FNAPs may be most beneficial or harmful to obesity risk; (3) the effects of FNAP policy and program changes, particularly around nutrition standards; and (4) where knowledge gaps (e.g., limited longitudinal data, FNAPs not assessed for obesity outcomes) still exist for the effectiveness of FNAPs in childhood obesity prevention. Given the high importance of childhood obesity to public health and health policy, understanding the degree to which nutrition assistance programs increase or decrease childhood obesity risk has important implications for future program design and implementation. This systematic review aims to fill this gap and address our guiding research question: what relationships have research studies found between nutrition assistance programs and children’s weight?

## 2. Materials and Methods

This systematic review followed established methods for conducting systematic reviews and reporting guidelines in the Preferred Reporting Items for Systematic Reviews and Meta-Analyses (PRISMA). A preliminary literature search suggested a breadth of studies around FNAPs, as well as previously conducted systematic reviews and meta-analyses examining various elements, settings, populations, interventions, and outcomes. Our research question met the established criteria warranting a systematic rather than a scoping review in its intention to address variation, investigate conflicting results, and inform future research [53]. A review by an Institutional Review Board was not relevant as the study did not involve human subjects in its design or execution [54].

We designed the search and study selection process to minimize potential bias in the research question development, search strategy and execution, identification of included articles, data extraction, and interpretation of findings. This was achieved through a combination of multiple reviewers, confirmation of extraction accuracy, and regular meetings to discuss and resolve questions. We submitted our study approach and design for PROSPERO registration during the exploratory phase and preliminary search process (CRD42022313675). The interdisciplinary research team represents diverse fields and intersecting interests in food security and health: public and social policy, public health sciences, social work, applied social and community psychology, and clinical psychology. The search strategy was developed with a School of Medicine research librarian. First, using PICO elements to construct a targeted systematic review of peer-review literature using scientific methods and quantitative data [55], we agreed on clear definitions of the problem and population, intervention, comparison, and outcome (Table 1).

The published literature was searched extensively using relevant search terms such as Food Supply, Food Assistance, Food Security, Body Mass Index, and Pediatric Obesity. The search strategy was conducted in PubMed/MEDLINE, ERIC (ProQuest), Elsevier EMBASE, SCOPUS, Clarivate Web of Science Core Collection (WOS), and The Cochrane Library from the inception of each database through December 2021 and again in July 2024. The final search results were uploaded to Covidence, an online tool for collaborative systematic review.

Two reviewers screened the title and abstract of each article. Each full-text article was reviewed independently by at least two reviewers with 97.8% interrater reliability. Interrater reliability was measured to identify any discrepancies in the reviewers’ interpretation of inclusion criteria for the screening and coding of articles. Reviewer conflicts were re-reviewed by multiple team members, discussed during weekly team meetings, and resolved by reaching consensus. Eligibility criteria included studies that focused on a U.S.-based nutrition assistance program with pediatric weight status or BMI reported as an outcome published in English-language peer-reviewed journals. Studies were excluded if they focused on adult outcomes, lacked an explicit study design (e.g., reviews, commentaries, or descriptive studies), or limited their presented findings to process outcome results (e.g., program uptake, participation, adherence, etc.). Additionally, descriptive studies that did not test for an effect or association between the intervention and weight outcome were excluded. The full-text review confirmed that all the included studies met these criteria.

In alignment with other recent reviews of food insecurity interventions, our search strategy targeted programs and policies that directly address a household’s ability to access, obtain, or afford sufficient food [47,48]. We expected our search to identify a range of studies examining governmental nutrition assistance programs. Initial literature searches revealed a few non-FNAP food security interventions or programs that reported on child weight. We reached the consensus not to include these programs since they were not focused on direct financial support or food provision in educational settings. In addition, while important for improving health, we excluded studies focused on nutritional counseling, environmental experiments, and fruit or vegetable consumption without an explicit voucher or subsidy component as these did not meet our definition of a nutrition assistance program.

Reviewers extracted data from the included articles, and assessed study quality and risk of bias using the Mixed Methods Appraisal Tool (MMAT) [56]. Across the included studies, we documented nutrition assistance programs, tests for any effect or association between the nutrition assistance program and pediatric weight, the study population, setting, sample size, design, comparison groups, weight variable(s), and whether weight variables were measured or self-reported. In addition, we documented whether studies framed weight change as a beneficial or adverse outcome, the study data source, funding source, and summary findings. Accounting for additional confounding variables was included as part of our study quality and risk-of-bias assessment. The presented findings primarily focus on weight, which was recorded for each study. In children aged 2 to 18, weight status is usually determined by BMI percentile based on the child’s sex and age. A BMI above the 85th percentile for age and sex is considered overweight while a BMI above the 95th percentile is considered obese [57]. For newborns and infants aged 0 to 2, weight is commonly measured as birthweight, weight-for-length, or weight-for-height.

We coded each study’s findings as identifying whether the nutrition assistance program had an adverse, beneficial, mixed, or no relationship with pediatric weight. Reviewers noted studies that focused on significant changes to the standard WIC package in 2009 or the implementation of the Healthy, Hunger-Free Kids Act of 2010 (HHFKA) on nutritional quality of school meals. Given the breadth of study designs, interventions, and variance in the presentation of the outcomes, summary and descriptive analysis were most appropriate for this review.

Finally, for article quality and risk-of-bias evaluation, we selected the MMAT to account for the range of study designs and methodologies across our sample. MMAT intends to deepen the descriptive and summary analysis in findings and comparisons rather than calculate and present cumulative scores. Five areas are rated as “Yes,” “No,” or “Can’t Tell.” Questions include whether the study sample represented the target population, whether the measurements were appropriate, whether the risk of nonresponse bias was low, and whether the statistical analyses were appropriate. Each article was assessed by a research team member and discussed during regular team meetings. An additional team member independently reviewed the five domains of the MMAT for 15 randomly selected articles to establish interrater reliability with 68 of 75 fields scored identically (91%).

As part of the study quality assessment and data extraction, we confirmed that positive and null findings were reflected and we documented study funding sources, a critical consideration for food and nutrition research [58]. Key areas assessed across articles included whether studies accounted for selection bias [59], as well as confounding variables since the relationships between FNAPs and weight can depend on myriad factors, including race and ethnicity [60], socioeconomic status [61], and level of food insecurity [62] FNAP-specific confounders, such as breast or formula-fed in relation to WIC and infant weight, parent-reported data [63], and participation in other FNAPs, were also evaluated.

## 3. Results

**Figure 1:**
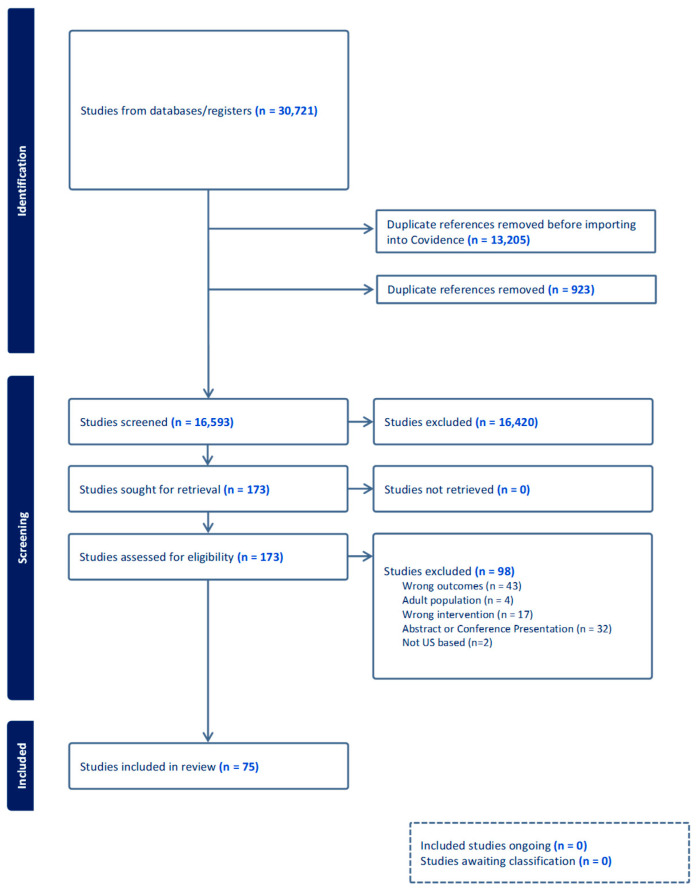
PRISMA Diagram of study selection for the Food Nutrition Assistance Programs and pediatric weight outcomes review.

As demonstrated in the PRISMA diagram (Figure 1), duplicate removal followed by title and abstract screening reduced the number of potentially eligible studies from 30,721 to 173 [64].

Most included studies (Table 2) were quantitative non-randomized studies that utilized cross-sectional or cohort designs. Following screening, we conducted a full-text review, which identified a final sample of 75 articles meeting the study inclusion criteria, of which 58 (77.33%) relied only on secondary survey or administrative data for their analyses, and 13 (17.33%) only used study-generated data [25,39,41,60,65,66,67,68,69,70,71,72,73,74,75,76,77,78,79,80,81,82,83,84,85,86,87,88,89,90,91,92,93,94,95,96,97,98,99,100,101,102,103,104,105,106,107,108,109,110,111,112,113,114,115,116,117,118,119,120,121,122,123,124,125,126,127,128,129,130,131,132,133,134,135]. Secondary data sources included the Early Childhood Longitudinal Study (ECLS), National Health and Nutrition Examination Survey (NHANES), Panel Study of Income Dynamics (PSID), and the National Longitudinal Survey of Youth (NLSY).

Among the included articles, 42 focused on FNAPs which provide financial support for families (19 SNAP, 21 WIC, and two both SNAP and WIC), 27 focused on FNAPs which provide food for children through educational settings (12 NSLP, five SBP, two CACFP, eight NSLP and SBP), and six examined multiple FNAPs (i.e., FNAPs providing financial support to families and FNAPs providing food through educational settings). Thirty-two studies found mixed results; 20 studies found a beneficial relationship between nutrition assistance programs and weight (i.e., the FNAP was associated with lower overweight and obesity, less frequent low birthweight); 16 found no effect or association; and seven found an adverse relationship.

Studies using secondary survey data were the most likely to find mixed results. Studies at a single clinic, city/county, or state were more likely to use primary study-generated data and report an adverse relationship or no associations. Studies with mixed results represented a range of populations and subgroups in which the suggested relationship between the intervention and weight did not hold. Study results varied by age, food insecurity status, or even which of the multiple FNAPs had a beneficial, adverse, or no relationship to weight. Most frequently, however, were sex-based differences in mixed study findings in 12 of the 32 studies (37.5%). Reflecting the diversity and transdisciplinary interest in food insecurity, studies were published in 45 unique peer-reviewed journals. Funding for the studies also came from multiple sources, concentrated in federal governmental and private foundation supports. Nineteen studies did not report a study funding source or support.

### 3.1. FNAPs Providing Food for Children Through Educational Settings (NSLP, SBP, CACFP)

#### 3.1.1. National School Lunch Program (NSLP)

Eleven studies focused on the NSLP, all of which used secondary data obtained through surveys. Seven of these studies only included kindergarten and elementary school-aged children, with four studies including children and adolescents in middle and high school. Ten studies measured weight outcomes and one used self-report. NSLP participation and weight relationships varied across studies. One study showed no association [67]. Two cross-sectional studies showed an adverse association in which participation in NSLP was associated with overweight and obesity [72,73]. Three cohort studies showed mixed associations across sex. For example, one study showed an adverse association for girls (NSLP participating girls had more rapid weight gain than non-NSLP participating girls), with neutral or no differences observed for boys [65]. Another study found a beneficial association for boys (3rd-grade boys had lower BMI growth than non-participants), with neutral or no differences observed for girls [68], and one study found an adverse association for boys but neutral or no association for girls [75]. Two cohort studies found mixed effects by HHFKA implementation. For example, Rothbart et al. found that the Community Eligibility Provision had a beneficial impact on weight outcomes, but only for second grade students (not other primary-grade students), and the effect was greatest for rural districts [74]. Three studies showed a beneficial association with obesity and overweight, one with a 17% reduction in obesity rates, another with a 6 percentage-point decrease in overweight and obesity, and one with a 2.4% reduction in obesity prevalence [66,69,76].

#### 3.1.2. School Breakfast Program (SBP)

Five studies assessed the SBP, one of which specifically looked at school breakfasts served in classrooms. All but one study focused on elementary and middle school students, and all studies had measured weight outcomes rather than self-report. Two studies found no association and three found mixed results for SBP and weight outcomes. Among studies finding mixed effects, a secondary data cohort study of 6495 children found mixed effects related to socioeconomic status. SBP was not independently associated with obesity; however, children (5th–8th grade) living below the federal poverty line who participated in SBP were twice as likely to develop obesity compared to children in low-income households who did not participate [78]. A second study, an RCT testing nutrition education and initiatives to increase SBP participation through breakfast in classrooms, found mixed effects by weight outcome [79]. The intervention was not associated with combined increases or decreases in overweight and obesity; however, it was associated with an increase in obesity alone. Authors suggested that breakfast in the classroom may contribute additional calories to dietary intake for children who are already overweight. A third study found mixed effects by initial BMI and sex [80]. In general, children who had an initial healthy weight were more likely to become overweight than children who were overweight developing obesity. The likelihood of moving to a higher BMI was greater among girls than boys.

#### 3.1.3. Combined NSLP and SBP

Eight studies looked at both NSLP and SBP. Four studies included kindergarten through high-school students; two focused on a single grade (4th grade, 8th grade); and two looked at elementary or middle school. Five studies were cross-sectional; three were cohort studies; and all but one used secondary data. Two studies, both cross-sectional, found no association with school meal participation and BMI [88,91]. Four studies found mixed associations. For example, one study found mixed association by sex (school meals associated with higher BMI scores for low-income girls but no effect was found for low-income boys), and another found mixed association by program (no association for NSLP and beneficial association for SBP, where additional school breakfasts were associated with lower BMI z-score) [85,89]. Two studies, one cross-sectional and one cohort, found adverse relationships with NSLP and SBP and weight outcomes. For example, Miller et al. found greater number of meals associated with higher BMI over time [87].

#### 3.1.4. Child and Adult Care Food Program (CACFP)

Only two studies assessed CACFP on weight outcomes, both of which found no association or effect. One study was a small cohort study of childcare centers serving predominantly Black children. No differences in weight were found between children attending CACFP-participating childcare centers and those at non-participating centers [82]. The second study was a cross-sectional study using secondary survey data of 4050 four-year-old children in childcare. No effect was found of CACFP participation on weight [83].

### 3.2. FNAPs Providing Financial Support for Families (SNAP and WIC)

#### 3.2.1. Supplemental Nutrition Assistance Program (SNAP)

Eighteen studies (11 cross-sectional, six cohort, and one cohort/cross-sectional) assessed SNAP, with five studies focused on children under six years of age. Most (*n* = 13) used secondary survey data, and four used study-collected data. Eight studies measured weight outcomes; five relied on self-report measures; and five incorporated both. Seven studies found mixed results, mostly due to difference across child sex and age. Four studies found a beneficial association between SNAP participation and weight; six studies found no association; and one study found an adverse relationship.

Among five studies finding a beneficial relationship between SNAP and weight, all were cross-sectional and focused on children ranging in age from two to 18 years old. Three studies found SNAP was associated with lower probability of being overweight, and one study found SNAP was associated with a 5.3% lower probability of developing obesity [25,115,121,122]. One cross-sectional study of 250 Black preschool children found a beneficial relationship in a reduction in underweight [111].

Among the nine mixed results studies, all used secondary survey data and five were cross-sectional. Three of these studies found mixed effects by sex and age for the outcomes of overweight and/or obesity. For example, among children aged five to 11 years old, participation in SNAP over the previous five years was associated with an increase in overweight for girls and a decrease in overweight for boys, with no association found for either sex for children ages 12–18 [112]. Three cross-sectional studies found mixed effects based on SNAP eligibility (e.g., higher BMI in SNAP recipients compared to other income groups but no difference in weight outcomes between SNAP recipients and income eligible non-recipients), enrollment date (e.g., children who enrolled in SNAP after the 2008 recession had lower BMIs compared to children who were previously enrolled but similar BMIs compared to those who never enrolled), and benefit level (e.g., SNAP was associated with a higher BMI when the benefit level was low but there was no association with BMI for higher benefit levels) [117,124,126]. One study found mixed effects by food security (food secure SNAP participants had higher BMI than food secure non-participants with no difference in BMI between food insecure SNAP participants and non-participants) [125].

One cross-sectional study that found an adverse relationship between participation and pediatric weight was conducted with 240 Hispanic children. The study found that WIC participants whose mothers reported receiving SNAP at any point were twice as likely to have high weight-for-length compared to mothers who never received SNAP [119].

#### 3.2.2. Women, Infants, and Children Supplemental Nutrition Assistance (WIC)

WIC had the highest proportion of studies finding a beneficial relationship between the intervention and weight, partly due to the 2009 WIC benefit package change that incentivized fruit and vegetable purchases. WIC studies were less frequent at the national level (5) and distinct in their use of governmental program administrative data. WIC studies overall had the largest sample sizes, and nearly all (19) used measured weight outcomes as opposed to self-report. The one self-report study was also one of two studies that found an adverse relationship between the intervention and weight [60]. Four studies included pregnant women and newborns; seven studies focused on children under three years; and 17 studies assessed children ages five and younger. Nine studies found a beneficial relationship; six reported mixed effects; two studies reported an adverse association; and four studies found no association (three assessed birthweight).

Among nine studies reporting a beneficial relationship, five were cohort studies and four assessed changes to WIC benefit package. Weight gain and obesity were the most common outcomes in these studies. For example, one cohort study of 182,618 children found that those who received a full dose of the new food package had a lower obesity risk at 4 years old compared to children receiving the old food package (RR = 0.88 for boys and 0.90 for girls) [102]. Two studies (one cohort, one cross-sectional) focused on birthweight and found beneficial outcomes of lower odds of low birthweight and increased birthweight [41,96]. Six studies found mixed effects across a variety of factors including race, sex, food environment, and infant feeding practices. For example, one study found that WIC increased mean birth weight among non-white participants by 177 g, and decreased rates of low birthweight by 3.8 percentage points, with no effects for white participants [95].

#### 3.2.3. Combined SNAP and WIC

Two studies assessed both WIC and SNAP. One study was a cross-sectional study using secondary data of 21,056 children aged two to 19 years from the 1970s–2002 [128]. This study found no association between WIC or SNAP and weight. The second study, a cross-sectional study of 66 infants from a single clinic, found a mixed association by program [129]. Women receiving SNAP were more likely to have an infant at risk of obesity; however, no associations were found between SNAP participation and risk for obesity.

### 3.3. Effects of FNAP Participation in Both Direct Financial and Food Through Educational Settings

Six studies looked at FNAPs, including both financial resources for families and provision of food for children in educational settings. All studies used secondary data, and sample sizes ranged from 500 to almost 5000 children. All but one study used cross-sectional study designs. Five of these studies looked at a combination of FNAPs rather than multiple FNAPs separately. One study found a beneficial association; three studies found mixed effects; and two studies found no association. No studies examining both financial resources and food provided through educational settings found an adverse relationship with pediatric weight. A study of 15–18-year-old adolescents found that participation in SNAP, NSLP, and SBP was associated with a 26.4% decrease in BMI [132]. Among studies finding mixed effects, one study of 1321 children and adolescents in low-income households found mixed results by food security [133]. Specifically, Kohn et al. found an adverse association with higher odds of high waist circumference and overweight or obesity for food secure children participating in two or more programs (SNAP, WIC, NSLP, SBP) but no adverse association with weigh circumference and overweight or obesity for food insecure children [133]. Another study of five-to-12-year-old children found that participation in SNAP, NSLP, and SBP was associated with lower odds of overweight in girls with food insecurity but found no association with lower odds of overweight for boys with food insecurity [130]. A third study found mixed results by program (e.g., SNAP participation was not associated with differences in the probability of being overweight; however, NSLP and SBP participation were associated with a higher probability of overweight) [131]. One study that found no association looked at whether SNAP and NSLP affected the relationship between food insecurity and BMI [134].

### 3.4. The Impact of Policy and Benefit Changes on Nutrition Assistance Programs

SNAP, WIC, and school meals have undergone a variety of changes since their inception. Our review identified 18 articles that specifically examined FNAP participation and weight in relation to notable policy changes that aimed to improve access or program impact on dietary intake and health such as a 2009 WIC Package Change, the HHFKA, and an increase in SNAP benefits. Eight studies found a significant beneficial relationship between the post-policy change FNAP and impacts on healthy weight outcomes, with nine studies having mixed results based on participant sex, income, rurality, age, and BMI, as well as food environment and policy implementation. No observable trends or shared characteristics differentiated studies that found mixed effects. One article found no association, and none found wholly adverse effects of the policy on pediatric weight.

### 3.5. Study Quality Assessment

Study quality assessment re-emphasized the homogeneity of the studies included in the review, as 73 (97.3%) studies used a quantitative non-randomized design. Forty-one included a cross-sectional study design; 27 used a cohort design; and seven presented both cross-sectional and longitudinal analyses and findings. Overall, 60 (80.0%) of the studies met the criteria for three or more of the five study quality assessment elements, 21 (28.0%) met four, and 16 (21.3%) fulfilled all five. Studies that met all five criteria represented a mix of interventions, cross-sectional and cohort designs, and summary weight outcomes and were mostly conducted at the U.S. national level. As a group, studies that found adverse relationships between FNAP participation and pediatric weight had lower study quality assessments in having three or less of the five criteria met.

## 4. Discussion

Food insecurity and obesity have short- and long-term consequences and adverse effects on children’s health [7,57]. Households participating in FNAPs are not homogeneous and should not be studied as such [132]. While no broad, universal finding emerged to indicate that participation in nutrition assistance programs causes adverse weight outcomes, 32 studies reported mixed results depending on subgroup (e.g., age, gender, or race/ethnicity). This suggests that nutrition assistance programs may play some role in a multifaceted and complex relationship with overweight and obesity. At the same time, the small number of studies either based in educational settings or direct financial support showing an adverse relationship between interventions and weight do not support claims that interventions are causing more harm or unintended consequences than good. Most studies found no relationship or a mixed or beneficial relationship between participation and overweight or obesity. These studies had higher quality assessments as they accounted for more confounding variables and more consistently used appropriate measures.

Across the literature, most studies reported mixed or null associations, and there were no observed trends from the sample that clearly indicated that any single FNAP or type (educational setting-based or financial support) reliably reduced or increased childhood overweight or obesity risk. Beneficial effects were observed across studies of different FNAPs, and, similarly, adverse findings were rare but present in both types. Studies of the WIC program had the largest proportion of beneficial findings, especially from evaluations of the 2009 package change. Sex was a frequent differentiator and subgroup moderator, especially for school meals. Other differences contributing to the complex relationships and frequency of mixed results included age, food security, combinations of programs, and different benefit levels.

We were also interested in determining whether specific ages during youth and adolescence would have more beneficial or adverse effects of FNAP participation. Again, our findings did not include any firm conclusions about the timing of FNAPs and their impacts, likely due to the broad and inconsistent age ranges used and the prevalence of cross-sectional study designs and not enough cohort studies that followed FNAP participants for long enough to assess timing. While some WIC studies found beneficial effects of participation in pregnancy and early childhood, SNAP and school meals studies that found mixed effects by age did not have clear patterns or trends.

Across FNAPs, the sample demonstrated methodological homogeneity, with the vast majority utilizing a quantitative non-randomized approach. Despite RCTs being the ‘gold standard’ for evidence, just two studies utilized a randomized control design. Other systematic reviews found few randomized studies for child-focused interventions or food insecurity interventions in health care settings [48]. Other recent reviews that focused on the charitable food system [51] and school meal consumption strategies [136] found proportionally more studies that utilized a randomized design [50]. Therefore, randomized designs are somewhat utilized around nutrition assistance programs and health, but not at the scale of the national policies and programs included in our results.

In addition to the homogeneity in study design and intervention type, we noted the study design and presentation of weight as a variable in findings across studies. While there is some acknowledgment in our sample and relevant adjacent systematic reviews surrounding weight or the limitations or shortcomings of BMI, most studies involving children and adolescents two years and older used BMI in some form (BMI, z-score, percentile, or interpreted as overweight or obesity) [82,111]. Studies that did not use BMI tended to involve young children either in relation to the WIC program or CACFP and used birthweight or weight-for-length.

Overall, nutrition assistance programs can play a critical role in improving outcomes and health during childhood [28,137]. From our study findings, while cognizant of mixed results from a variety of settings and designs, the potential for broad negative unintended consequences on children’s weight from nutrition assistance programs appears to be low. At the same time, of the 18 studies that examined the impact of an FNAP policy change, for example, to expand benefit amounts or update intervention nutritional standards, eight found beneficial relationships, nine found mixed, one found no relationship, and no studies identified an adverse, positive relationship between participation in the updated FNAP pediatric weight. This demonstrates that policy changes strengthening nutrition standards and improving benefit adequacy such as the WIC package change and HHFKA may have an important benefit for program participant weight moving in healthy directions. No evidence suggested that these improvements or expansions to FNAPs worsened obesity risk at the population level, and mixed results likely reflect heterogeneity rather than ineffectiveness or unintended consequences.

Our systematic review found that the studies with adverse positive relationships between weight change and FNAPs generally had lower study quality assessments and tended to be conducted either at the national or single site level rather than the state or local level. While these represent a mix of cross-sectional and cohort design studies, they presented study quality issues, such as not identifying or accounting for whether the intervention occurred as intended across comparison groups, reliance upon self- or parent-reported weight or intervention participation, and the use of inadequate participation metrics (e.g., school meal participation represented by if any meal was consumed by any household member over a 12-month period). Significant study dropout was also noted (as high as 54% in one study), and, for school meals, the study design suggested a more foundational and widely acknowledged finding that children who are eligible for nutrition assistance programs are more likely to have indicators of overweight and obesity. Notably, studies that found a beneficial relationship where the intent was to decrease underweight also typically had lower quality assessment scores.

Our study has several strengths to highlight. By constructing a comprehensive search strategy, achieving high interrater reliability, and having a clear, collaborative process for resolving conflicts, our final sample is representative of the literature directly relevant to our research question. The MMAT framework made it possible to summarize study quality across our sample, and a test for interrater reliability found significant consistency and a minimal risk of reviewer bias. Our systematic review also has limitations. Despite utilizing a replicable process and achieving an important level of interrater reliability, relevant studies may have been inadvertently excluded during the screening process or missed by the online database searches. While we checked for commonly cited studies in our final sample, we did not conduct comprehensive backward and forward reference searches. BMI and related measures are imprecise indicators of nutritional status, though they are often the most collected and reported. Given their prevalence and associations with long-term health outcomes, BMI-related measures were important for examination. Lastly, the broad range of study outcomes did not allow for a meta-analysis of findings beyond descriptive and observational trends.

While challenges certainly confront both FNAPs and associated research in trying to address and understand complex nuances of food insecurity, weight, and program participation, a few key knowledge gaps would benefit from future examination, in particular, longitudinal data, expanded evaluations of programs such as CACFP or multiple FNAP participation within and across educational settings and financial assistance, and greater emphasis and incorporation of implementation fidelity, program variation, and local contextual factors. Enhanced understanding of the complexities of food insecurity, obesity, and the programs that comprise hundreds of billions of dollars annually and significant food and meals consumed can inform future policy. There is significant value in improving access to nutritious, high-quality diets as the association between food insecurity and overweight has been attributed to lower nutritional quality and higher-calorie diets consumed [138]. Policy discussions of nutrition assistance programs have highlighted the risk of causing harm, for example, alleging that universal school breakfast leads students to double their breakfasts and consume significantly more calories than needed for healthy growth and development. An unintended consequence [139] of these interventions, then, could be the risk of an increase in overweight and obesity to the extent that it would be more harmful than providing hungry children with increased access to food at school. Conversely, nutrition assistance programs may have a positive effect on obesity and weight status. Research suggests that the regularity and consistency of meals can impact metabolism such that irregular or inconsistent food access leads to increased weight gain [140]. As such, programs and policies that provide children with regular food access, such as school breakfast or lunch, may be especially consequential.

## 5. Conclusions

This systematic review did not find evidence of any broad direct adverse relationship between FNAP participation and overweight or obesity in educational settings or through direct financial assistance. While referenced as “supplemental,” SNAP and WIC, alongside other educational setting-based food and nutrition programs such as the NSLP and SBP, can constitute a significant part of dietary intake, especially for infants, children, and adolescents. The co-occurrence in some studies demonstrating mixed or even adverse relationships between food insecurity, obesity, and FNAP participation is worthy of consideration surrounding root causes and continued improvements and innovations to food environments, health system, and myriad interventions aiming to support healthy, thriving communities. At the same time, finding no conclusive evidence that FNAPs have broad adverse effects on pediatric weight outcomes across 75 studies spanning multiple decades combined with encouraging results from studies examining expansions or nutritional standard updates to FNAPs can inform and encourage health, education, and other policy decision makers to continue to expand and improve nutritional quality and access through these programs.

## Figures and Tables

**Table 1:** PICO Framework.

PICO Element	Considerations
P (problem, patient, population)	Household food insecurity, overweight and obesity; children and adolescents 0 to 18 years of age
I (intervention)	Food insecurity policy and program interventions that increase access to food
C (comparison)	Individuals not receiving the intervention (when possible, eligible and non-eligible)
O (outcome)	Child weight status (i.e., underweight, overweight, obesity, Body Mass Index (BMI))

**Table 2:** Summary of Included Studies.

Study	Population	Setting	Study Design	Weight Outcome	Relationship	Data Source
**Food Nutrition Assistance Programs in Education Settings**						
**National School Lunch Program (NSLP)**						
Hernandez 2011 [65]	1140 Kindergarteners	U.S.	Cohort	BMI %	Mixed effects: Sex	ECLS
Gundersen 2012 [66]	2693 6–17-year-olds	U.S.	Cohort	Obesity	Beneficial association	NHANES; Administrative Data
Oza-Frank 2013 [67]	33,672 3rd graders	State	Cohort	BMI %; Overweight; Obesity	No association	Study Data
Vericker 2019 * [68]	14,720 kindergarten, 1st and 3rd graders	U.S.	Cohort	BMI z-score	Mixed associations: Sex	ECLS
Jia 2020 * [69]	9172 K to 12th graders	U.S.	Cohort	BMI %; BMI % category	Beneficial effect	NHANES
Andreyeva 2021 * [70]	2500 K to 5th graders	U.S.	Cohort	BMI %; BMI z-score; Overweight; Obesity	Mixed effects: Income	ECLS-K
Richardson 2022 * [71]	5958 K to 5th graders	U.S.	Cohort	BMI %	Mixed effects: HHFKA implementation	ECLS-K
Chavez 2023 [72]	15,136 0–18-year-olds	U.S.	Cross-sectional	BMI %	Adverse association	US Census Bureau National Survey of Children’s Health (NSCH)
Sang 2023 [73]	21,678 6th graders	State	Cross-sectional	BMI %; Overweight; Obesity	Adverse association	Project Healthy Schools
Rothbart 2023 * [74]	K to 10th graders	State	Cohort	BMI %; Overweight; Obesity	Mixed effects: Grade; Rurality; Implementation	NYS Education Department, NYS School Report Card, NYS Department of Health (NYSDOH)
Bonomo 2024 [75]	K to 5th graders	U.S.	Cohort	BMI; Overweight; Obesity	Mixed effects: Sex; Socioeconomic status	ECLS-K; School Nutrition Dietary Assessment (SNDA)
Localio 2024 * [76]	5th, 7th, 9th graders	State	Cross-sectional	BMI %;	Beneficial association	CDE and the National Center for Education Statistics
**School Breakfast Program (SBP)**						
Corcoran 2016 [77]	860,000 K to 8th graders	City/ County	Cross-Sectional	BMI z-score; Obesity	No association	Administrative Data
Sudharsanan 2016 [78]	6495 1st–8th graders	U.S.	Cohort	Obesity	Mixed effect: SES	ECLS
Polonsky 2019 [79]	1362 4th–6th graders	City/ County	RCT	BMI z-score; BMI % category	Mixed associations: Breakfast in Classroom Intervention	Study Data
Abouk 2022 [80]	6310 K to 5th graders	U.S.	Cohort	Overweight; Obesity	Mixed effects: BMI; Sex	ECLS-K
Bullock 2022 [81]	K to 12th graders	U.S.	Longitudinal	BMI %	No association	
**Child and Adult Care Food Program (CACFP)**						
Bruening 1999 [82]	40 3–5-year-olds	U.S.	Cohort	Weight-for-age; Height-for-age; Weight-for-height	No association	Study Data
Korenman 2013 [83]	4050 4-year-olds	U.S.	Cross-Sectional	BMI %; BMI % category	No effect	ECLS
**Combined NSLP and SBP**						
Vermeersch 1984 [84]	6553 6–18-year-olds	U.S.	Cross-Sectional	Weight-for-age; Weight-for-height	Mixed association: Age	National Evaluation of School Nutrition Programs
Gleason 2009 [85]	2228 1st–12th graders	U.S.	Cross-Sectional	BMI %; BMI z-score; Overweight; Obesity	Mixed associations: Program	School Nutrition Dietary Assessment Study
Li 2010 [86]	62,880 6–17-year-olds	U.S.	Cross-Sectional	BMI	Adverse association	National Survey of Children’s Health
Miller 2011 [87]	11,400 K to 5th graders	U.S.	Cohort	BMI	Adverse effect	ECLS
Paxton 2012 [88]	1535 4th graders	City/ County	Cross-Sectional	BMI %	No association	Study Data
Vericker 2014 [89]	1550 8th graders	U.S.	Cohort	BMI; Obesity	Mixed associations: Sex	ECLS
Capogrossi 2017 [90]	14,710 1st–8th graders	U.S.	Cohort	BMI z-score; Overweight	Mixed effects: Region, Rurality, Program	ECLS
Bardin 2021 [91]	1963 6–19-year-olds	U.S.	Cross-Sectional	BMI; Overweight; Obesity	No association	School Nutrition and Meal Cost Study
**Direct Financial Support**						
**Special Supplemental Nutrition Program for Women, Infants, and Children (WIC)**						
Heimendinger 1984 [92]	1907 0–18-month-olds	City/ County	Cohort	Weight z-Scores	Beneficial effect	Administrative Data
Collins 1985 [93]	519 Pregnant women and newborns	State	Cross-Sectional	Birthweight	No association	Study Data; Health Records
Metcoff 1985 [94]	824 Pregnant women and newborns	State	RCT	Birthweight	Mixed associations: Smoking Status	Study Data
Stockbauer 1986 [95]	6732 Pregnant women and newborns	State	Cross-Sectional	Birthweight	Mixed effects: Race	Administrative Data
Caan 1987 [96]	703 0–3-year-olds	State	Cohort	Birthweight; Birth length; Low birthweight	Beneficial association	Study Data
Rush 1988 [97]	3782 Pregnant women and newborns	U.S.	Cohort	Birthweight	No association	Study Data
Black 2004 [98]	5923 0–36-month-olds	Multi-City/ County	Cross-Sectional	Weight-for-age z-score; Length-for-age z-score; Overweight; Caregiver perception of overweight	No association	Study Data
Melgar-Quiñonez 2004 [99]	204 3–5-year-olds	State	Cross-Sectional	Overweight; Obesity	Beneficial association	Study Data
Hoynes 2011 [41]	18,517 Infants	U.S.	Cross-Sectional	Birthweight	Beneficial effect	Administrative Data
Edmunds 2014 [100]	157,590 0–12-month-olds	State	Cohort	Weight Gain	Beneficial association	Administrative Data
Aldrich 2016 [60]	31,294 6–24-month-olds	U.S.	Cross-Sectional	Weight-for-length	Adverse association	Administrative Data
Chaparro 2019 ** [101]	106,150 0–4-year-olds	City/ County	Cohort	Weight-for-height z-score; BMI z-score; Obesity	Mixed effects: Sex; BMI	Administrative Data
Chaparro 2019 ** [102]	182,618 0–4-year-olds	City/ County	Cohort	Weight-for-height z-score; BMI z-score; Obesity	Beneficial association	Administrative Data
Daepp 2019 ** [103]	2.25 million 2–4-year-olds	U.S.	Cross-Sectional	Obesity	Beneficial effect	Administrative Data; Previous Study Data
Anderson 2020 ** [104]	149,634 0–4-year-olds	City/ County	Cohort	Weight-for-height z-score; Obesity	Mixed effects: Sex; Food environment	Administrative Data
Chaparro 2020 ** [105]	74,821 0–4-year-olds	City/ County	Cohort	Weight-for-height z-score; Obesity	Beneficial effect	Administrative Data
Chaparro 2020 ** [106]	116,991 0–4-year-olds	City/ County	Cohort	Weight-for-height z-score; Obesity	Mixed effects: Sex; breastfeeding	Administrative Data
Chaparro 2021 ** [107]	79,502 4-year-olds	City/ County	Cross-Sectional	Weight-for-height z-score; Obesity	Beneficial effect	Administrative Data
Anderson 2022 [108]	836 0–9-year-olds	U.S.	Cohort	BMI	Adverse association	WIC Infant and Toddler Feeding Practices Study Data
Wang 2022 [109]	1,831,649	State	Quasi experimental	Birthweight	No association	Birth certificate data from National Center for Health Statistics
Anderson 2024 [110]	59,132 0–4-year-olds	State	Cohort	BMI z-score	Mixed effects: Race, Food environment, Formula	WIC program data from large agency in Los Angeles County
**Supplemental Nutrition Assistance Program (SNAP)**						
Kafatos 1977 [111]	250 0–6-year-old Black children	City/ County	Cross-Sectional	Weight percentile	Beneficial effect	Study Data
Gibson 2004 [112]	7843 5–18-year-olds	U.S.	Cohort	Overweight; Obesity	Mixed associations: Sex; Age	NLSY
Gibson 2006 [113]	3594 4.5–11.5-year-old daughters	U.S.	Cohort	BMI; Obesity	No association	NLSY
Robinson 2011 [114]	1147 5–18-year-olds	U.S.	Cohort	BMI	Mixed effects: Sex; Age	NLSY
Burgstahler 2012 [25]	360 2–18-year-olds	Multi-State	Cross-Sectional	Overweight	Beneficial association	Survey of Household Finances and Childhood Obesity
Kreider 2012 [115]	4418 2–17-year-olds	U.S.	Cross-Sectional	Obesity	Beneficial effect	NHANES
Schmeiser 2012 [39]	16,553 5–18-year-olds	U.S.	Cohort	Overweight; Obesity	Mixed effects: Sex; Age	NLSY
Simmons 2012 [116]	386 3–4-year-olds	State	Cross-sectional & cohort	BMI %	No effect	Study Data
Leung 2013 [117]	5193 4–19-year-olds	U.S.	Cross-Sectional	BMI z-score	No association	NHANES
Fan 2015 [118]	More than 5200 12–20-year-olds	U.S.	Cohort	BMI; Obesity	No effect	NLSY
Barroso 2016 [119]	240 12–24-month-old Hispanic WIC-enrolled children	Single Clinic	Cross-Sectional	Weight-for-length	Adverse association	Study Data
Leung 2017 [120]	4450 12–19-year-olds	U.S.	Cross-Sectional	BMI z-score	No association	NHANES
Gorski Findling 2018 [121]	3748 2–18-year-olds	U.S.	Cross-Sectional	Overweight	Beneficial association	Food Acquisition and Purchase Survey
Ettinger de Cuba 2019 [122]	28,782 0–2-year-olds	Multi-City/ County	Cross-Sectional	At risk for obesity; Underweight	Beneficial association	Children’s Health Watch Study Data
Foster 2019 [123]	52 3–5-year-olds	City/ County	Cross-Sectional	BMI z-score	No association	Study Data
Hamad 2019 [124]	4548 0–17-year-olds	U.S.	Cross-Sectional	BMI	Mixed associations: SNAP Enrollment Date	PSID
Lee 2019 [125]	2662 12–19-year-olds	U.S.	Cross-Sectional	BMI z-score	Mixed associations: Food Security	NHANES
Reynolds 2020 [126]	5289 5–18-year-olds	U.S.	Cohort	BMI	Mixed effects: SNAP Benefit Level	PSID; Administrative Data
Hudak 2021 [127]	1570 pre-ARRA and 2505 post-ARRA 2–18-year-olds	U.S.	Cross-Sectional	BMI %; BMI z-score; BMI % category	Mixed associations: Age	NHANES
**Combined WIC and SNAP**						
VerPloeg 2008 [128]	21,056 2–19-year-olds	U.S.	Cross-Sectional	BMI; Overweight; Obesity	No association	NHANES
Watt 2013 [129]	66 0–12-month-olds	Single Clinic	Cross-Sectional	Weight-for-length	Mixed associations: Program	Study Data
**Multiple [Intervention Type]**						
Jones 2003 [SNAP, School Meals] [130]	772 5–12-year-olds	U.S.	Cross-Sectional	Overweight	Mixed effects: Sex; Food insecurity	PSID
Hofferth 2005 [SNAP, School Meals] [131]	1268 6–12-year-olds	U.S.	Cross-Sectional	Obesity	Mixed associations: Program	PSID
Roy 2012 [SNAP, School Meals] [132]	518 15–18-year-olds	U.S.	Cross-Sectional	BMI; Obesity	Beneficial association	American Time Use Survey
Kohn 2014 [SNAP, School Meals, WIC] [133]	1321 4–17-year-olds	U.S.	Cross-Sectional	BMI z-score	Mixed effects: Food Security	NHANES
Nguyen 2017 [SNAP, School Meals] [134]	4719 9–17-year-olds	U.S.	Cross-Sectional	BMI %	No association	NHANES
Lee 2022 [SNAP, School Meals] [135]	4457 K to 5th graders	U.S.	Cohort	BMI z-score	No association	ECLS-K

* indicates a study of the Healthy, Hunger-Free Kids Act (HHFKA), ** indicates a WIC Package Change study.

## Data Availability

Data are available by request to the corresponding author.

## Abbreviations

The following abbreviations are used in this manuscript:

CACFP	Child and Adult Care Food Program
FNAP	Federal Nutrition Assistance Program
HHFKA	Healthy Hunger-Free Kids Act
MMAT	Mixed Methods Appraisal Tool
NSLP	National School Lunch Program
SBP	School Breakfast Program
SNAP	Supplemental Nutrition Assistance Program
WIC	Special Supplemental Nutrition Program for Women, Infants, and Children

## References

[1] Coleman-Jensen A., Rabbitt M.P., Gregory C.A., Singh A. (2021). Household Food Security in the United States in 2020.

[2] Rabbitt M.P., Hales L.J., Burke M.P., Coleman-Jensen A. (2023). Household Food Security in the United States in 2022.

[3] Engelhard E., Hake M. (2020). Food Security Evidence Review: Key Drivers and What Works to Improve Food Security.

[4] Kaur J., Lamb M.M., Ogden C.L. (2015). The association between food insecurity and obesity in children—The National Health and Nutrition Examination Survey. J. Acad. Nutr. Diet..

[5] Warren M., West M., Beck S. (2023). The State of Obesity: Better Policies for a Healthier America 2023.

[6] Stierman B., Afful J., Carroll M.D., Chen T.C., Davy O., Fink S., Fryar C.D., Gu Q., Hales C.M., Hughes J.P. (2021). National Health and Nutrition Examination Survey 2017—March 2020 Prepandemic Data Files—Development of Files and Prevalence Estimates for Selected Health Outcomes.

[7] Singh A.S., Mulder C., Twisk J.W.R., Van Mechelen W., Chinapaw M.J.M. (2008). Tracking of childhood overweight into adulthood: A systematic review of the literature. Obes. Rev..

[8] Simmonds M., Llewellyn A., Owen C.G., Woolacott N. (2016). Predicting adult obesity from childhood obesity: A systematic review and meta-analysis. Obes. Rev..

[9] Brisbois T.D., Farmer A.P., McCargar L.J. (2012). Early markers of adult obesity: A review. Obes. Rev..

[10] Waters H., Graf M. (2018). America’s Obesity Crisis: The Health and Economic Costs of Excess Weight.

[11] St. Pierre C., Ver Ploeg M., Dietz W.H., Pryor S., Jakazi C.S., Layman E., Noymer D. (2022). Food insecurity and childhood obesity: A systematic review. Pediatrics.

[12] Tester J.M., Rosas L.G., Leung C.W. (2020). Food insecurity and pediatric obesity: A double whammy in the era of COVID-19. Curr. Obes. Rep..

[13] Dinour L.M., Bergen D., Yeh M.C. (2007). The food insecurity-obesity paradox: A Review of the literature and the role food stamps may play. J. Am. Diet. Assoc..

[14] Crawford P.B., Webb K.L. (2011). Unraveling the paradox of concurrent food insecurity and obesity. Am. J. Prev. Med..

[15] Myers C.A., Mire E.F., Katzmarzyk P.T. (2020). Trends in Adiposity and Food Insecurity among US Adults. JAMA Netw. Open.

[16] Hooper L., Telke S., Larson N., Mason S.M., Neumark-Sztainer D. (2020). Household food insecurity: Associations with disordered eating behaviours and overweight in a population-based sample of adolescents. Public Health Nutr..

[17] Drewnowski A., Darmon N. (2005). The economics of obesity: Dietary energy density and energy cost. Am. J. Clin. Nutr..

[18] Lopez R.P. (2007). Neighborhood risk factors for obesity. Obesity.

[19] Nettle D., Andrews C., Bateson M. (2017). Food insecurity as a driver of obesity in humans: The insurance hypothesis. Behav. Brain Sci..

[20] Dhurandhar E.J. (2016). The food-insecurity obesity paradox: A resource scarcity hypothesis. Physiol. Behav..

[21] Smith T.A., Berning J.P., Yang X., Colson G., Dorfman J.H. (2016). The Effects of Benefit Timing and Income Fungibility on Food Purchasing Decisions among Supplemental Nutrition Assistance Program Households. Am. J. Agric. Econ..

[22] Cassady D.L., Liaw K., Miller L.M.S. (2015). Disparities in Obesity-related outdoor advertising by neighborhood income and race. J. Urban Health.

[23] Seligman H.K., Laraia B.A., Kushel M.B. (2010). Food insecurity is associated with chronic disease among low-income NHANES participants. J. Nutr..

[24] Franklin B., Jones A., Love D., Puckett S., Macklin J., White-Means S. (2012). Exploring mediators of food insecurity and obesity: A review of recent literature. J. Community Health.

[25] Burgstahler R., Gundersen C., Garasky S. (2012). The Supplemental Nutrition Assistance Program, financial stress, and childhood obesity. Agric. Resour. Econ. Rev..

[26] Mabli J., Ohls J. (2015). Supplemental Nutrition Assistance Program participation is associated with an increase in household food security in a national evaluation. J. Nutr..

[27] Metallinos-Katsaras E., Gorman K.S., Wilde P., Kallio J. (2011). A longitudinal study of WIC participation on household food insecurity. Matern. Child Health J..

[28] Cohen J.F.W., Hecht A.A., Mcloughlin G.M., Turner L., Schwartz M.B., Varela-Moreiras G. (2021). Universal school meals and associations with student participation, attendance, academic performance, diet quality, food security, and body mass index: A systematic review. Nutrients.

[29] King M.D., Giefer K.G. (2021). Most Children Receiving SNAP Get at Least One Other Social Safety Net Benefit.

[30] Oliveira V., Racine E., Olmsted J., Ghelfi L.M. (2002). The WIC Program: Background, Trends, and Issues.

[31] U.S. Department of Agriculture Food and Nutrition Service (2025). Updates to the School Nutrition Standards.

[32] Hayes C., FitzSimons C. (2025). The Reach of School Breakfast and Lunch During the 2023–2024 School Year.

[33] Jones J.W., Todd J.E., Toossi S. (2025). The Food and Nutrition Assistance Landscape: Fiscal Year 2024 Annual Report.

[34] Seligman H.K., Berkowitz S.A. (2019). Aligning programs and policies to support food security and public health goals in the United States. Annu. Rev. Public Health.

[35] Meyerhoefer C.D., Pylypchuk Y. (2008). Does participation in the food stamp program increase the prevalence of obesity and health care spending?. Am. J. Agric. Econ..

[36] Baum C.L. (2012). The effects of food stamp receipt on weight gained by expectant mothers. J. Popul. Econ..

[37] Fox M., Hamilton W., Lin B. (2004). Effects of Food Assistance and Nutrition Programs on Nutrition and Health: Volume 3, Literature Review.

[38] Schanzenbach D.W. (2009). Do school lunches contribute to childhood obesity?. J. Human. Resour..

[39] Schmeiser M.D. (2012). The impact of long-term participation in the supplemental nutrition assistance program on child obesity. Health Econ..

[40] Dietz W.H. (2021). Better diet quality in the Healthy Hunger-Free Kids Act and WIC package reduced childhood obesity. Pediatrics.

[41] Hoynes H., Page M., Stevens A.H. (2011). Can targeted transfers improve birth outcomes? Evidence from the introduction of the WIC program. J. Public Econ..

[42] Andreyeva T., Tripp A.S., Schwartz M.B. (2015). Dietary quality of Americans by Supplemental Nutrition Assistance Program participation status: A systematic review. Am. J. Prev. Med..

[43] Schultz D.J., Byker Shanks C., Houghtaling B. (2015). The Impact of the 2009 Special Supplemental Nutrition Program for Women, Infants, and Children Food Package Revisions on Participants: A Systematic Review. J. Acad. Nutr. Diet..

[44] Koleilat M., Whaley S.E., Esguerra K.B., Sekhobo J.P. (2017). The Role of WIC in Obesity Prevention. Curr. Pediatr. Rep..

[45] Simonovich S.D., Pineros-Leano M., Ali A., Awosika O., Herman A., Withington M.H., Loiacono B., Cory M., Estrada M., Soto D. (2020). A systematic review examining the relationship between food insecurity and early childhood physiological health outcomes. Transl. Behav. Med..

[46] Jaime P.C., Lock K. (2009). Do school based food and nutrition policies improve diet and reduce obesity?. Prev. Med..

[47] Oronce C.I.A., Miake-Lye I.M., Begashaw M.M., Booth M., Shrank W.H., Shekelle P.G. (2021). Interventions to Address Food Insecurity Among Adults in Canada and the US. JAMA Health Forum.

[48] De Marchis E.H., Torres J.M., Benesch T., Fichtenberg C., Allen I.E., Whitaker E.M., Gottlieb L.M. (2019). Interventions addressing food insecurity in health care settings: A systematic review. Ann. Fam. Med..

[49] Spoede E., Corkins M.R., Spear B.A., Becker P.J., Bellini S.G., Hoy M.K., Piemonte T.A., Rozga M. (2021). Food Insecurity and Pediatric Malnutrition Related to Under- and Overweight in the United States: An Evidence Analysis Center Systematic Review. J. Acad. Nutr. Diet..

[50] Holley C.E., Mason C. (2019). A systematic review of the evaluation of interventions to tackle children’s food insecurity. Curr. Nutr. Rep..

[51] An R., Wang J., Liu J., Shen J., Loehmer E., McCaffrey J. (2019). A systematic review of food pantry-based interventions in the USA. Public Health Nutr..

[52] Winterton R., Warburton J., Oppenheimer M. (2013). The future for Meals on Wheels? Reviewing innovative approaches to meal provision for ageing populations. Int. J. Soc. Welf..

[53] Munn Z., Peters M.D.J., Stern C., Tufanaru C., McArthur A., Aromataris E. (2018). Systematic review or scoping review? Guidance for authors when choosing between a systematic or scoping review approach. BMC Med. Res. Methodol..

[54] U.S. Department of Health & Human Services (2020). Office for Human Research Protections. Human Subject Regulations Decision Charts: 2018 Requirements.

[55] Stern C., Jordan Z., Mcarthur A. (2014). Developing the review question and inclusion criteria. Am. J. Nurs..

[56] Hong Q.N., Fàbregues S., Bartlett G., Boardman F., Cargo M., Dagenais P., Gagnon M.P., Griffiths F., Nicolau B., O’Cathain A. (2018). The Mixed Methods Appraisal Tool (MMAT) version 2018 for information professionals and researchers. Educ. Inf..

[57] Apovian C.M. (2016). Obesity: Definition, comorbidities, causes, and burden. Am. J. Manag. Care.

[58] Nestle M. (2016). Corporate funding of food and nutrition research science or marketing?. JAMA Intern. Med..

[59] Story M. (2009). The Third School Nutrition Dietary Assessment Study: Findings and Policy Implications for Improving the Health of US Children. J. Am. Diet. Assoc..

[60] Aldrich H., Gance-Cleveland B. (2016). Comparing weight-for-length status of young children in two infant feeding programs. Matern. Child Health J..

[61] Singh G.K., Siahpush M., Kogan M.D. (2010). Rising Social Inequalities in US Childhood Obesity, 2003–2007. Ann. Epidemiol..

[62] Rose D., Bodor J.N. (2006). Household food insecurity and overweight status in young school children: Results from the early childhood longitudinal study. Pediatrics.

[63] Paxton-Aiken A.E., Baxter S.D., Tebbs J.M., Finney C.J., Guinn C.H., Royer J.A. (2012). How accurate are parental responses concerning their fourth-grade children’s school-meal participation, and what is the relationship between children’s body mass index and school-meal participation based on parental responses?. Int. J. Behav. Nutr. Phys. Act..

[64] Page M.J., McKenzie J.E., Bossuyt P.M., Boutron I., Hoffmann T.C., Mulrow C.D., Shamseer L., Tetzlaff J.M., Moher D. (2021). Updating guidance for reporting systematic reviews: Development of the PRISMA 2020 statement. J. Clin. Epidemiol..

[65] Hernandez D.C., Francis L.A., Doyle E.A. (2011). National school lunch program participation and sex differences in body mass index trajectories of children from low-income families. Arch. Pediatr. Adolesc. Med..

[66] Gundersen C., Kreider B., Pepper J. (2012). The impact of the National School Lunch Program on child health: A nonparametric bounds analysis. J. Econometr..

[67] Oza-Frank R., Hade E.M., Norton A., Scarpitti H., Conrey E.J. (2013). Trends in Body Mass Index among Ohio’s Third-Grade Children: 2004–2005 to 2009–2010. J. Acad. Nutr. Diet..

[68] Vericker T.C., Gearing M.E., Kim S.D. (2019). Updated Nutrition Standards for School Meals Associated with Improved Weight Outcomes for Boys in Elementary School. J. Sch. Health.

[69] Jia J., Moore L.L., Cabral H., Hanchate A., LaRochelle M.R. (2020). Changes to dietary and health outcomes following implementation of the 2012 updated US Department of Agriculture school nutrition standards: Analysis using National Health and Nutrition Examination Survey, 2005–2016. Public Health Nutr..

[70] Andreyeva T., Sun X. (2021). Universal school meals in the US: What can we learn from the community eligibility provision?. Nutrients.

[71] Richardson A.S., Weden M.M., Cabreros I., Datar A. (2022). Association of the Healthy, Hunger-Free Kids Act of 2010 with Body Mass Trajectories of Children in Low-Income Families. JAMA Netw. Open.

[72] Chavez L., Malik N., Kapella-Mshigeni S. (2023). The National School Lunch Program and Obesity: A Look at Economic Stability’s Influence on the Relationship. J. Sch. Health.

[73] Sang C.J., de Visser R., Krallman R., Pai C.W., Montgomery D., Moser C.A., Kline-Rogers E., DuRussel-Weston J., Eagle K.A., Chinapaw M. (2023). Cardiometabolic Risk and Dietary Behaviors in Middle-School Children Consuming School-Sourced Lunch. Acad. Pediatr..

[74] Rothbart M.W., Schwartz A.E., Gutierrez E. (2023). Paying for free lunch: The impact of CEP universal free meals on revenues, spending, and student health. Educ. Financ. Policy.

[75] Bonomo T., Schanzenbach D.W. (2024). Trends in the school lunch program: Changes in selection, nutrition & health. Food Policy.

[76] Localio A.M., Knox M.A., Basu A., Lindman T., Walkinshaw L.P., Jones-Smith J.C. (2024). Universal Free School Meals Policy and Childhood Obesity. Pediatrics.

[77] Corcoran S.P., Elbel B., Schwartz A.E. (2016). The Effect of Breakfast in the Classroom on Obesity and Academic Performance: Evidence from New York City. J. Policy Anal. Manag..

[78] Sudharsanan N., Romano S., Cunningham S.A. (2016). School Breakfast Receipt and Obesity among American Fifth- and Eighth-Graders. J. Acad. Nutr. Diet..

[79] Polonsky H.M., Bauer K.W., Fisher J.O., Davey A., Sherman S., Abel M.L., Hanlon A., Ruth K.J., Dale L.C., Foster G.D. (2019). Effect of a Breakfast in the Classroom Initiative on Obesity in Urban School-aged Children: A Cluster Randomized Clinical Trial. JAMA Pediatr..

[80] Abouk R., Adams S. (2022). Breakfast After the Bell: The Effects of Expanding Access to School Breakfasts on the Weight and Achievement of Elementary School Children. Econ. Educ. Rev..

[81] Bullock S.L., Dawson-Mcclure S., Truesdale K.P., Ward D.S., Aiello A.E., Ammerman A.S. (2022). Associations between a Universal Free Breakfast Policy and School Breakfast Program Participation, School Attendance, and Weight Status: A District-Wide Analysis. Int. J. Environ. Res. Public Health.

[82] Bruening K.S., Gilbride J.A., Passannante M.R., McClowry S. (1999). Dietary intake and health outcomes among young children attending 2 urban day-care centers. J. Am. Diet. Assoc..

[83] Korenman S., Abner K.S., Kaestner R., Gordon R.A. (2013). The Child and Adult Care Food Program and the nutrition of preschoolers. Early Child. Res. Q..

[84] Vermeersch J., Hanes S., Gale S. (1984). The National Evaluation of School Nutrition Programs: Program impact on anthropometric measures. Am. J. Clin. Nutr..

[85] Gleason P.M., Dodd A.H. (2009). School Breakfast Program but Not School Lunch Program Participation Is Associated with Lower Body Mass Index. J. Am. Diet. Assoc..

[86] Li J.I., Hooker N.H. (2010). Childhood Obesity and Schools: Evidence from the National Survey of Children’s Health. J. Sch. Health.

[87] Miller D.P. (2011). Associations between the home and school environments and child body mass index. Soc. Sci. Med..

[88] Paxton A.E., Baxter S.D., Tebbs J.M., Royer J.A., Guinn C.H., Devlin C.M., Finney C.J. (2012). Nonsignificant relationship between participation in school-provided meals and body mass index during the fourth-grade school year. J. Acad. Nutr. Diet..

[89] Vericker T.C. (2014). Children’s school-related food and physical activity behaviors are associated with body mass index. J. Acad. Nutr. Diet..

[90] Capogrossi K., You W. (2017). The Influence of School Nutrition Programs on the Weight of Low-Income Children: A Treatment Effect Analysis. Health Econ..

[91] Bardin S., Gola A.A. (2021). Analyzing the association between student weight status and school meal participation: Evidence from the school nutrition and meal cost study. Nutrients.

[92] Heimendinger J., Laird N., Austin J., Timmer P., Gershoff S. (1984). The effects of the WIC program on the growth of infants. Am. J. Clin. Nutr..

[93] Collins T., DeMellier S., Leeper J., Milo T. (1985). Supplemental Food Program: Effects on Pregnancy Outcomes. South. Med. J..

[94] Metcoff J., Costiloe P., Crosby W.M., Dutta S., Sandstead H.H., Milne D., Bodwell C.E., Majors S.H. (1985). Effect of food supplementation (WIC) during pregnancy on birth weight. Am. J. Clin. Nutr..

[95] Stockbauer J. (1986). Evaluation of the Missouri WIC program: Prenatal components. J. Am. Diet. Assoc..

[96] Caan B., Horgen D.M., Margen S., King J.C., Jewell N.P. (1987). Benefits associated with WIC supplemental feeding during the interpregnancy interval. Am. J. Clin. Nutr..

[97] Rush D., Leighton J., Sloan N.L., Alvir J.M., Horvitz D.G., Seaver W.B., Garbowski G.C., Johnson S.S., Kulka R.A., Devore J.W. (1988). The National WIC Evaluation: Evaluation of the Special Supplemental Food Program for Women, Infants, and Children. VI. Study of infants and children. Am. J. Clin. Nutr..

[98] Black M.M., Cutts D.B., Frank D.A., Geppert J., Skalicky A., Levenson S., Casey P.H., Berkowitz C., Zaldivar N., Cook J.T. (2004). Special Supplemental Nutrition Program for Women, Infants, and Children participation and infants’ growth and health: A multisite surveillance study. Pediatrics.

[99] Melgar-Quiñonez H.R., Kaiser L.L. (2004). Relationship of child-feeding practices to overweight in low-income Mexican-American preschool-aged children. J. Am. Diet. Assoc..

[100] Edmunds L.S., Sekhobo J.P., Dennison B.A., Chiasson M.A., Stratton H.H., Davison K.K. (2014). Association of prenatal participation in a public health nutrition program with healthy infant weight gain. Am. J. Public Health.

[101] Chaparro M.P., Anderson C.E., Crespi C.M., Whaley S.E., Wang M.C. (2019). The effect of the 2009 WIC food package change on childhood obesity varies by gender and initial weight status in Los Angeles County. Pediatr. Obes..

[102] Chaparro M.P., Crespi C.M., Anderson C.E., Wang M.C., Whaley S.E. (2019). The 2009 special supplemental nutrition program for Women, Infants, and Children (WIC) food package change and children’s growth trajectories and obesity in Los Angeles County. Am. J. Clin. Nutr..

[103] Daepp M.I.G., Gortmaker S.L., Claire Wang Y., Long M.W., Kenney E.L. (2019). WIC food package changes: Trends in childhood obesity prevalence. Pediatrics.

[104] Anderson C.E., Crespi C.M., Wang M.C., Whaley S.E., Chaparro M.P. (2020). The neighborhood food environment modifies the effect of the 2009 WIC food package change on childhood obesity in Los Angeles County, California. BMC Public Health.

[105] Chaparro M.P., Anderson C.E., Crespi C.M., Wang M.C., Whaley S.E. (2020). The new child food package is associated with reduced obesity risk among formula fed infants participating in the Special Supplemental Nutrition Program for Women, Infants and Children (WIC) in Los Angeles County, California, 2003–2016. Int. J. Behav. Nutr. Phys. Act..

[106] Chaparro M.P., Wang M.C., Anderson C.E., Crespi C.M., Whaley S.E. (2020). The Association between the 2009 WIC Food Package Change and Early Childhood Obesity Risk Varies by Type of Infant Package Received. J. Acad. Nutr. Diet..

[107] Chaparro M.P., Whaley S.E., Anderson C.E., Wang M.C., Crespi C.M. (2021). The role of income and neighbourhood poverty in the association between the 2009 Special Supplemental Nutrition Program for Women, Infants and Children (WIC) food package change and child obesity among WIC-participating children in Los Angeles County, 2003–2016. Public Health Nutr..

[108] Anderson C.E., Martinez C.E., Ritchie L.D., Paolicelli C., Reat A., Borger C., Whaley S.E. (2022). Longer Special Supplemental Nutrition Program for Women, Infants, and Children (WIC) Participation Duration Is Associated with Higher Diet Quality at Age 5 Years. J. Nutr..

[109] Wang G., Seligman H., Levi R., Hamad R. (2022). Impact of fruit and vegetable benefits on pregnancy outcomes among WIC participants: A natural experiment. Transl. Behav. Med..

[110] Anderson C.E., Whaley S.E., Goran M.I. (2024). The neighborhood food environment modifies the association between infant feeding and childhood obesity. BMC Public Health.

[111] Kafatos A.G., Zee P. (1977). Nutritional Benefits from Federal Food Assistance: A Survey of Preschool Black Children from Low-Income Families in Memphis. Am. J. Dis. Child..

[112] Gibson D. (2004). Long-Term Food Stamp Program Participation is Differentially Related to Overweight in Young Girls and Boys. J. Nutr..

[113] Gibson D. (2006). Long-Term Food Stamp Program Participation Is Positively Related to Simultaneous Overweight in Young Daughters and Obesity in Mothers. J. Nutr..

[114] Robinson C.A., Zheng X. (2011). Household Food Stamp Program Participation and Childhood Obesity. J. Agric. Resour. Econ..

[115] Kreider B., Pepper J.V., Gundersen C., Jolliffe D. (2012). Identifying the effects of SNAP (Food Stamps) on child health outcomes when participation is endogenous and misreported. J. Am. Stat. Assoc..

[116] Simmons S., Alexander J.L., Ewing H., Whetzel S. (2012). SNAP Participation in Preschool-Aged Children and Prevalence of Overweight and Obesity. J. Sch. Health.

[117] Leung C.W., Blumenthal S.J., Hoffnagle E.E., Jensen H.H., Foerster S.B., Nestle M., Cheung L.W., Mozaffarian D., Willett W.C. (2013). Associations of food stamp participation with dietary quality and obesity in children. Pediatrics.

[118] Fan M., Jin Y. (2015). The Supplemental Nutrition Assistance Program and Childhood Obesity in the United States: Evidence from the National Longitudinal Survey of Youth 1997. Am. J. Health Econ..

[119] Barroso C.S., Roncancio A., Moramarco M.W., Hinojosa M.B., Davila Y.R., Mendias E., Reifsnider E. (2016). Food security, maternal feeding practices and child weight-for-length. Appl. Nurs. Res..

[120] Leung C.W., Tester J.M., Rimm E.B., Willett W.C. (2017). SNAP Participation and Diet-Sensitive Cardiometabolic Risk Factors in Adolescents. Am. J. Prev. Med..

[121] Gorski Findling M.T., Wolfson J.A., Rimm E.B., Bleich S.N. (2018). Differences in the Neighborhood Retail Food Environment and Obesity Among US Children and Adolescents by SNAP Participation. Obesity.

[122] Ettinger de Cuba S.A., Bovell-Ammon A.R., Cook J.T., Coleman S.M., Black M.M., Chilton M.M., Casey P.H., Cutts D.B., Heeren T.C., Sandel M.T. (2019). SNAP, Young Children’s Health, and Family Food Security and Healthcare Access. Am. J. Prev. Med..

[123] Foster J.S., Adamsons K., Vollmer R.L., Mobley A.R. (2019). A pilot study of low-income mothers and fathers of preschool age children to determine the relationship of food security and nutrition assistance on feeding style and child body weight. J. Hunger. Environ. Nutr..

[124] Hamad R., Templeton Z.S., Schoemaker L., Zhao M., Bhattacharya J. (2019). Comparing demographic and health characteristics of new and existing SNAP recipients: Application of a machine learning algorithm. Am. J. Clin. Nutr..

[125] Lee A.M., Scharf R.J., Filipp S.L., Gurka M.J., Deboer M.D. (2019). Food Insecurity Is Associated with Prediabetes Risk among U.S. Adolescents, NHANES 2003–2014. Metab. Syndr. Relat. Disord..

[126] Reynolds M.M., Fox A.M., Wen M., Varner M.W. (2020). Is less more? Examining the relationship between food assistance benefit levels and childhood weight. SSM Popul. Health.

[127] Hudak K.M., Racine E.F. (2021). Do additional SNAP benefits matter for child weight? Evidence from the 2009 benefit increase. Econ. Hum. Biol..

[128] Ver Ploeg M., Mancino L., Lin B.H., Guthrie J. (2008). US Food assistance programs and trends in children’s weight. Int. J. Pediatr. Obes..

[129] Watt T.T., Appel L., Roberts K., Flores B., Morris S. (2013). Sugar, stress, and the Supplemental Nutrition Assistance Program: Early childhood obesity risks among a clinic-based sample of low-income Hispanics. J. Community Health.

[130] Jones S.J., Jahns L., Laraia B.A., Haughton B. (2003). Lower Risk of Overweight in School-aged Food Insecure Girls Who Participate in Food Assistance: Results from the Panel Study of Income Dynamics Child Development Supplement. Arch. Pediatr. Adolesc. Med..

[131] Hoefferth S.L., Curtin S. (2005). Poverty, food programs, and childhood obesity. J. Policy Anal. Manag..

[132] Roy M., Millimet D.L., Tchernis R. (2012). Federal nutrition programs and childhood obesity: Inside the black box. Rev. Econ. Househ..

[133] Kohn M.J., Bell J.F., Grow H.M.G., Chan G. (2014). Food insecurity, food assistance and weight status in US youth: New evidence from NHANES 2007–08. Pediatr. Obes..

[134] Nguyen B.T., Ford C.N., Yaroch A.L., Shuval K., Drope J. (2017). Food Security and Weight Status in Children: Interactions with Food Assistance Programs. Am. J. Prev. Med..

[135] Lee M.M., Kinsey E.W., Kenney E.L. (2022). U.S. Nutrition Assistance Program Participation and Childhood Obesity: The Early Childhood Longitudinal Study 2011. Am. J. Prev. Med..

[136] Cohen J.F.W., Hecht A.A., Hager E.R., Turner L., Burkholder K., Schwartz M.B. (2021). Strategies to improve school meal consumption: A systematic review. Nutrients.

[137] Gundersen C., Ziliak J.P. (2015). Food insecurity and health outcomes. Health Aff..

[138] Ludwig D.S., Blumenthal S.J., Willett W.C. (2012). Opportunities to Reduce Childhood Hunger and Obesity. J. Am. Med. Assoc..

[139] Oliver K., Lorenc T., Tinkler J. (2019). Evaluating unintended consequences: New insights into solving practical, ethical and political challenges of evaluation. Evaluation.

[140] Alhussain M.H., Macdonald I.A., Taylor M.A. (2016). Irregular meal-pattern effects on energy expenditure, metabolism, and appetite regulation: A randomized controlled trial in healthy normal-weight women. Am. J. Clin. Nutr..

